# Quantifying erosion-induced carbon emissions from SOC decomposition across sediment pathways in the yellow river basin

**DOI:** 10.1186/s13021-025-00380-7

**Published:** 2026-01-07

**Authors:** Jinwei Guo, Yuchun Yang, Mukesh Kumar Soothar, Yanbing Qi

**Affiliations:** 1 Shanxi Provincial Department-Municipal Key Laboratory Cultivation Base for Quality Enhancement and Utilization of Shangdang Chinese Medicinal Materials, School of Pharmacy, Changzhi Medical College, Changzhi, 046000 Shanxi China; 2 College of Natural Resources and Environment, Northwest A&F University, Yangling, 712100 Shaanxi China

**Keywords:** Soil erosion, Sediment transfer, SOC decomposition rate, Carbon emission, Yellow river basin

## Abstract

**Background:**

Soil erosion not only leads to soil loss but also redistributes soil organic carbon (SOC) and releases carbon dioxide (CO_2_) that contributes significantly to regional carbon emissions. Great efforts have been made to prevent soil erosion in the Yellow River Basin (YRB) in China in the past decades. Only few studies have paid attention to carbon emissions from soil loss. This study integrates the China Soil Loss Equation (CSLE) with a transport-limited sediment delivery (TLSD) model to quantify sediment redistribution and associated carbon emissions across five depositional processes (slope, reservoir, plain, river channel, and regional output) in the YRB.

**Results:**

The CSLE-TLSD model calibrated to a significantly improved Nash-Sutcliffe efficiency of 0.5690, compared to 0.5628 for the CSLE model. Results reveal that 28.50 ± 4.43% of eroded SOC was decomposed during transport, releasing 2.48 ± 0.11 × 10^8^ t CO_2_ in the YRB from 1990 to 2020. Striking spatial disparities emerged in different regions: the upper reaches exhibited a SOC decomposition ratio of 49.66 ± 4.40%, in sharp contrast to 22.96 ± 10.35% in the middle reaches. The five provinces with the highest carbon emission rate from 1990 to 2020 were Shanxi (15.45 t CO_2_/km^2^), Shaanxi (14.23 t CO_2_/km^2^), Shandong (13.10 t CO_2_/km^2^), Qinghai (11.98 t CO_2_/km^2^), and Gansu (11.25 t CO_2_/km^2^).

**Conclusion:**

These findings underscore the necessity of incorporating erosion-driven carbon flux dynamics into terrestrial carbon accounting frameworks, particularly in basins undergoing intensive anthropogenic modification.

**Supplementary Information:**

The online version contains supplementary material available at 10.1186/s13021-025-00380-7.

## Background

Terrestrial ecosystems play a vital role in addressing the challenges of global climate change [1]. As the largest carbon pool in the terrestrial ecosystem [2], soil regulates atmospheric carbon dioxide (CO_2_) concentrations and climate change [3]. Soil erosion leads to the redistribution of sediment and the organic matter it carries. These sediments are transported by rivers and deposited in low-lying areas of the basin or transported to rivers and marine ecosystems [4]. The major portion of soil organic carbon (SOC) consists of active carbon, which is more prone to mineralization and release during sediment transport caused by soil erosion [5]. This process increased the risk of SOC loss [6], leading to higher CO_2_ emissions [7] and further reducing SOC storage [8, 9]. Among the various relevant surface processes that affect the dynamics of atmospheric CO_2_, soil erosion has emerged as an important variable [9, 10]. Therefore, the contribution of SOC loss to atmospheric CO_2_ due to soil erosion should not be underestimated.

Soil erosion is a complex process involving multiple spatiotemporal scales [11], and its carbon flux effect depends on the interaction between erosion and deposition. During the process of sediment transport, the uncertainty regarding the sources, fate, and quantity of SOC has led to ongoing debates about the “carbon source/sink” role of soil erosion [12]. Although previous studies have developed models to simulate soil erosion processes [13], existing models still face limitations in accurately capturing the continuous erosion-sedimentation process, particularly in accounting for the effects of reservoir interception and floodplain deposition on sediment dynamics, thereby posing challenges to regional carbon cycle research. Therefore, a comprehensive assessment of sediment and organic carbon redistribution across various depositional processes, along with a refined estimation of carbon emissions induced by soil erosion, is essential for advancing our understanding of carbon cycling and organic carbon dynamics in terrestrial and fluvial systems.

In the Yellow River Basin (YRB), serious soil erosion has been causing significant harm to land productivity and people’s survival [14, 15], due to its fragile ecosystem, erodible soil, concentrated rainstorms, dense gullies, and intensive agricultural activities [16]. Because of the prolonged, continuous and acute soil erosion, the destruction of topsoil exposes the subsoil, leading to an increased risk of CO_2_ emissions [17, 18], and resulting in substantial carbon flux between the soil and the atmosphere. However, the model deficiencies hinder the effective quantification of sediment deposition processes associated with large-scale reservoir construction in the YRB (e.g., key projects such as the Xiaolangdi Dam) and major sediment accumulation areas such as the Hetao Plain and the Fenwei Plain.

Current studies on SOC redistribution have increasingly benefited from the long-term, multi-scale developments in soil erosion and sedimentology. Among various models, the China Soil Loss Equation (CSLE) has been widely applied at national, regional, and watershed scales [19–21], demonstrating particularly good adaptability and representativeness in the YRB and its subregions [22–24]. The integration of erosion models with the Transport-Limited Sediment Delivery (TLSD) model has shown significant improvements in prediction accuracy, enabling more detailed simulations of sediment redistribution across different geomorphic units [25]. In recent years, several studies have demonstrated the applicability of this integrated approach across diverse geographic contexts, such as in the Yarlung Tsangpo River Basin [26], the YRB [27], and the upper reaches of the YRB [28].

However, while plot or hillslope scale studies provide detailed process understanding, they often fail to capture the complete sediment pathway from source to sink. In contrast, larger-scale assessments tend to oversimplify the complex interactions among erosion, transport, and deposition, neglecting the spatial heterogeneity that governs sediment connectivity. Therefore, a basin-scale analysis is essential, as it represents an integrated geomorphic unit encompassing the full continuum of soil detachment and sediment on slopes, sediment transport along river networks, and final deposition in major sinks such as reservoirs and plains. The CSLE–TLSD model is structurally designed to address this gap: the CSLE component quantifies hillslope erosion, while the TLSD explicitly simulates sediment redistribution and deposition along flow pathways based on topographic convergence and transport-limiting conditions. This integration enables a process-based, spatially explicit estimation of sediment and carbon fluxes across the source-to-sink continuum, providing a robust foundation for evaluating erosion-induced carbon redistribution at the basin scale.

Based on the above, there are three objectives in this study: (1) Analysis of the characteristics of sediment distribution in different regions of the YRB; (2) Analysis of the characteristics of SOC in different regions of the YRB; (3) Estimation of the carbon emission from sediment generated by soil erosion in the YRB. To address these questions, this study employed the CSLE with the TLSD model to estimate soil erosion and sediment redistribution in the YRB, which form the basis for subsequent carbon emission assessments. We used the spatial distribution data of soil erosion in the YRB and sediment transport data from 30 main hydrological stations and 28 tributary hydrological stations from 1988 to 2012 to analyze the redistribution of erosion sediment across five processes—slope sedimentation, reservoir sedimentation, river channel sedimentation, plain sedimentation, and regional sediment output—for the entire YRB. We further investigated the carbon flux between soil and the atmosphere during the erosion-sedimentation process in the YRB from 1990 to 2020 by analyzing SOC data. The research objectives were to provide data support and theoretical insights for understanding organic carbon cycling under the influence of soil erosion.

## Materials and methods

### Study area

**Fig. 1:**
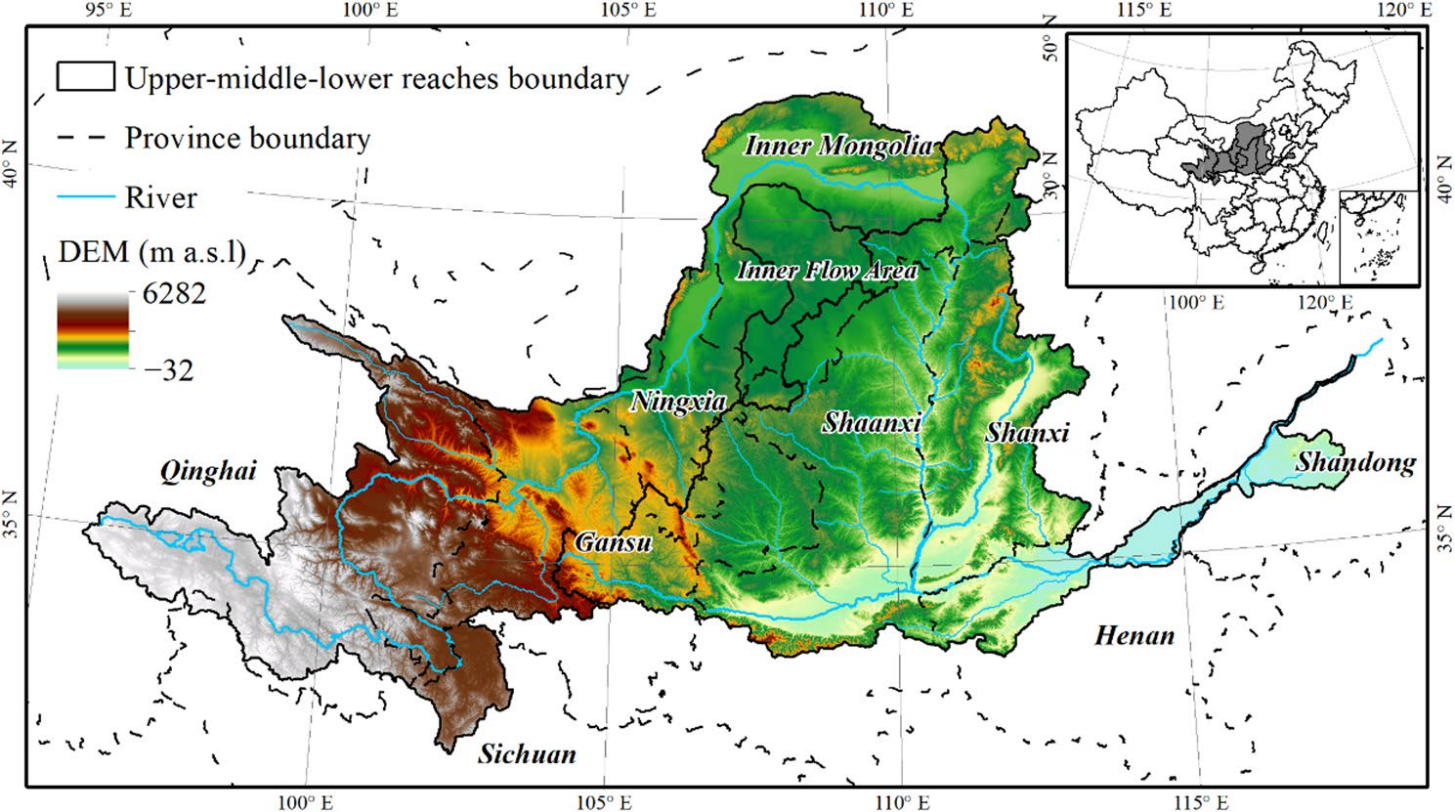
Location of study area

The Yellow River flows through nine provinces including Qinghai, Sichuan, Gansu, Ningxia, Inner Mongolia, Shanxi, Shaanxi, Henan, and Shandong (Fig. 1). The YRB covers a total area of 79.5 × 10^4^ km^2^, with a latitude ranging from 32°10′ to 41°50′ N and a longitude ranging from 95°53′ to 119°05′ E [29]. The study area is roughly composed of the upper, middle, and lower reaches according to the hydrological and geographical characteristics [30].

## Data collection and pre-processing

**Fig. 2:**
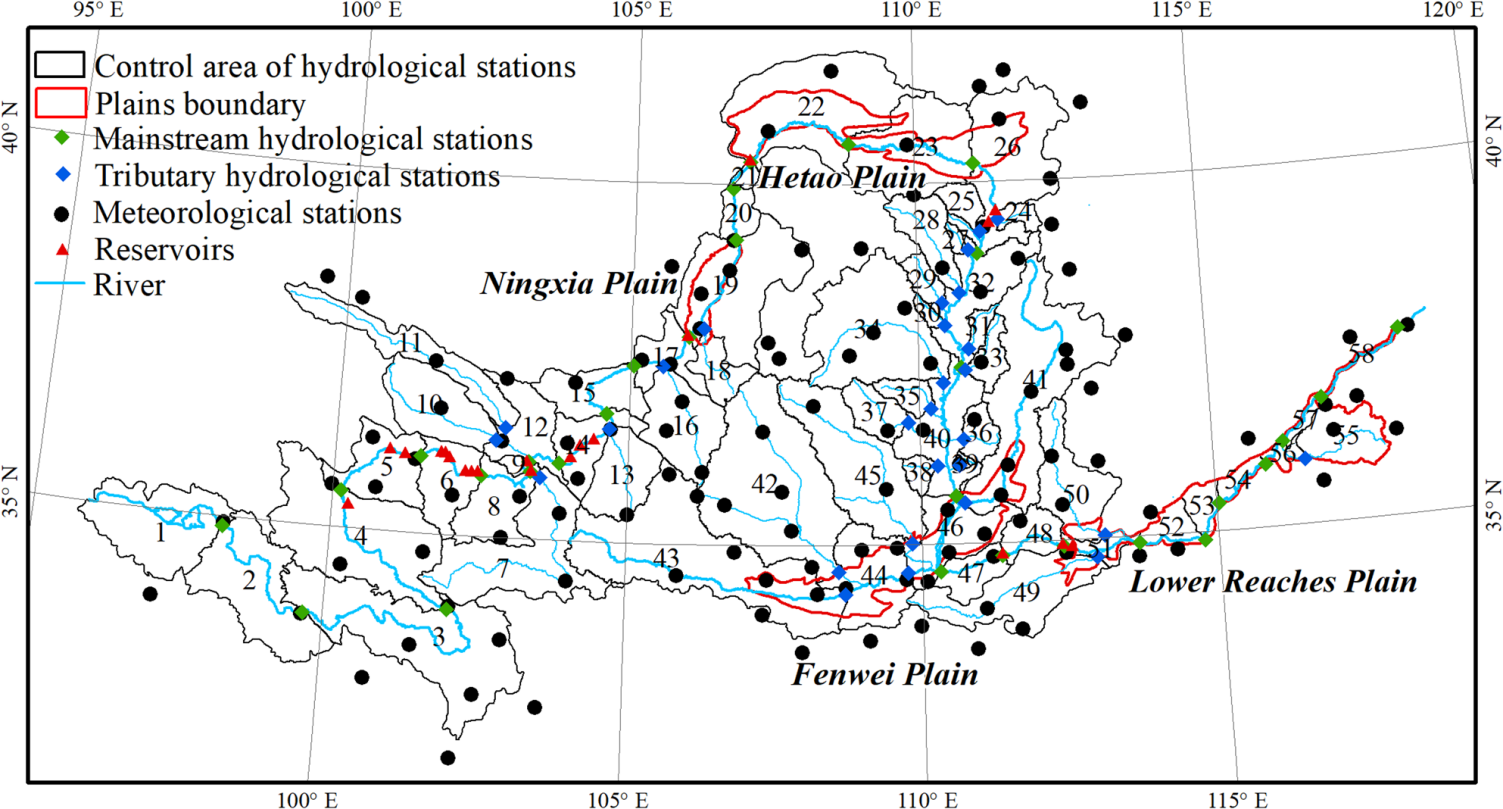
Spatial distribution of hydrological stations and their control areas, meteorological stations, reservoirs and plain areas

Non-spatial data and spatial data were used in this study. Non-spatial data included hydrological and meteorological data. The meteorological data included daily precipitation data from 1988 to 2022 from 122 national meteorological stations (Fig. 2) within the YRB [31], used to collect the rainfall erosivity factor (R) of (CSLE) model. The hydrological data included annual sediment transport data from 30 mainstream hydrological stations and 28 tributary hydrological stations within the YRB from 1988 to 2012, as well as the commissioning dates of 21 mainstream reservoirs (Fig. 2 and Tables S1, S2). The annual sediment transport data were sourced from the Yellow River Water and Sediment Spatiotemporal Atlas (Second Edition) [32], which were collected from 30 mainstream hydrological stations and 28 tributary hydrological stations distributed in the YRB region. Due to the lack of sediment accumulation data for most reservoirs, sedimentation volumes were estimated using hydrological station data.

The spatial data constituted land use data from 1990 to 2020 with a spatial resolution of 30 m [33]; Landsat vegetation coverage data with a spatial resolution of 30 m from 1990 to 2020 (Google Earth Engine platform); Digital elevation model (DEM) with a spatial resolution of 30 m [34]; Soil erodibility factor (K) data with a spatial resolution of 30 m [35]; Spatial distribution data of horizontal terracing in China for 2018 [36] and in the Loess Plateau for the years 2000, 2010, and 2015 with a spatial resolution of 30 m [37]; Layered data with six layers (0–5 cm、5–15 cm、15–30 cm、30–60 cm、60–100 cm and100-200 cm) of SOC content and soil bulk density for the YRB with a spatial resolution of 90 m [38]. Due to the limitation of computer data processing capability, all data in this study were resampled to a resolution of 500 m.

## Methods

### Estimating sediment distribution

**Fig. 3:**
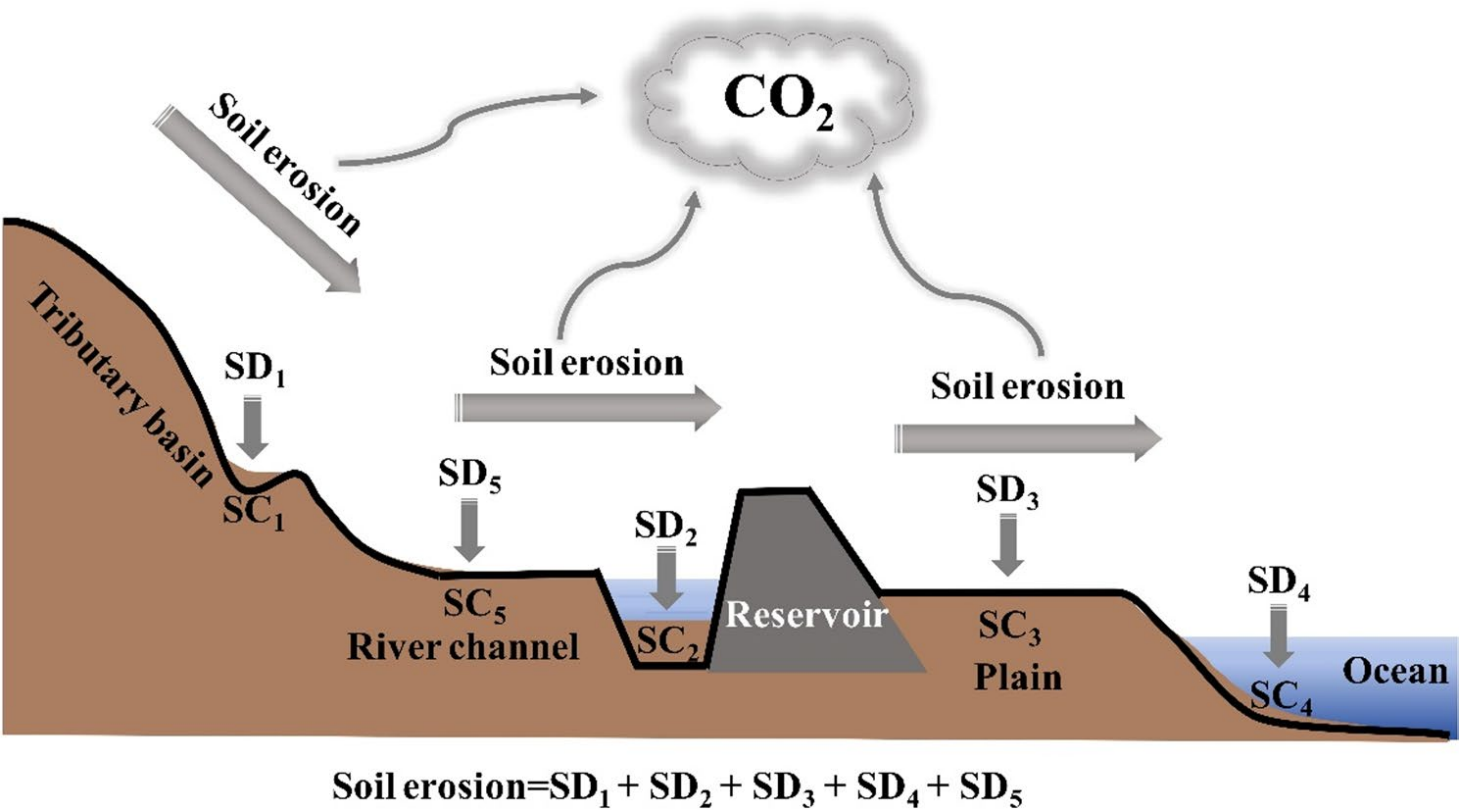
A conceptual diagram showing the production, transport, and deposition of sediment and organic carbon within a river basin. SD_1_, SD_2_, SD_3_, SD_4_, SD_5_ are the sediment deposition amounts (t) in slope, reservoirs, plains, regional outputs, and river channels, respectively; SC_1_, SC_2_, SC_3_, SC_4_, SC_5_ are the SOC content in slope, reservoirs, plains, regional outputs, and river channels, respectively

The soil erosion at the basin scale usually consists of three stages, including the separation, transportation, and deposition of eroded soil particles, it is a redistribution process of soil movement and deposition under the action of external forces [39]. After soil erosion occurs, the main redistribution processes include slope sedimentation, reservoir sedimentation, river channel sedimentation, plain sedimentation, and regional sediment output (Fig. 3). Therefore, we estimated sediment redistribution as well as carbon emissions caused by soil erosion based on the five sedimentary processes in the YRB region.

**(Ⅰ) Potential soil erosion.** Potential soil erosion was estimated by the CSLE, with the inclusion of a gully erosion factor [40] (Eq. 1):

1 $$\:A=R\times\:K\times\:LS\times\:B\times\:E\times\:T\times\:G$$

Where *A* is the average soil erosion modulus(t∙hm^−2^∙yr^−1^); *R* is the rainfall erosivity factor (MJ∙mm/(hm^2^∙h∙a)); *K* is the soil erodibility factor (t∙hm^2^∙h/(hm^2^∙MJ∙mm)); *LS* is the slope length and slope factor; *B* is the biological practice factor; *E* is the engineering practice factor; *T* is the tillage measures factor and *G* is the gully erosion factor. The calculation methods for *R*, *LS*, *B*, *E*, and *T* factors referred to the study by [41] and [24].

**(Ⅱ)Slope sedimentation (SD**_**1**_**).** The sedimentation on the slope (SD₁), which represents the first and most immediate sink for potential soil erosion (A), was calculated using the TLSD model. The sediment transport capacity was calculated by Eq. 2 [24].

2 $$\:TC={K}_{TC}\times\:R\times\:K\times\:{A}_{s}^{1.44}\times\:{S}_{3D}^{1.44}$$

where *TC* is the sediment transport capacity (t∙hm^−2^∙yr^−1^); *K*_*TC*_ is the transport capacity coefficient; *A*_*s*_ is the specific catchment area contributed by the upslope per unit contour length (m^2^∙m^−1^); *S*_*3D*_ is the local slope for three-dimensional landscapes (m∙m^−1^). *K*_*TC*_ was estimated by Eq. 3 [42].

3 $$\:{K}_{TC}=\beta\:\times\:exp\left(-NDVI/1-NDVI\right)$$

where *β* is the calibration coefficient, which is used to adjust the error between the observed and predicted sediment yield.

The sediment deposition amount was calculated by Eqs. 4 and 5 [43].

4 $$\:{T}_{out}=min\left(A+\sum\:{T}_{in},TC\right)$$

5 $$\:{SD}_{1}=A+\sum\:{T}_{in}-{T}_{out}$$

where *T*_*in*_ is the sediment inflow in the current cell from upstream cells; *T*_*out*_ is the sediment outflow from the current cell; *A* is potential soil erosion modulus in the current cell (t∙hm^−2^∙yr^−1^); *SD*_*1*_ is sediment deposition modulus in the current cell (t∙hm^−2^∙yr^−1^).

The net soil erosion, which denotes the actual soil loss from the slope after initial deposition, is calculated as the difference between the potential soil erosion and the slope sedimentation (Eq. 6).

6 $$\:NE=A-{SD}_{1}$$

where *NE* is the net soil erosion modulus (t∙hm^−2^∙yr^−1^).

**(Ⅲ)Reservoir sedimentation (SD**_**2**_**).** The sediment deposition amount of the reservoirs in each interval was calculated by summing the sediment input from the hydrological stations in the upper reaches, sediment input from tributary basins, and the interval soil erosion amount, and then subtracting the sediment output at the lower reaches hydrological station. To account for the time lag between reservoir construction and actual sediment accumulation, the commissioning date (i.e., the year when each reservoir began water storage) was used as the basis for defining the sediment accumulation period. Specifically, sediment deposition calculations for each interval began one year after the reservoir’s commissioning date to allow for the initiation of sediment trapping. The time window for sediment accumulation thus varied across different intervals depending on reservoir start times (Table S3). A total of 21 reservoirs in the YRB involved 12 hydrological station control intervals, with 17 reservoirs in 8 hydrological station control intervals in the upper reaches and 5 reservoirs in 4 hydrological station control intervals in the middle reaches (Table S3).

**(Ⅳ) Plain sedimentation (SD**_**3**_**).** The sediment deposition amount in plains was calculated by summing the sediment input from the hydrological stations in upper reaches, sediment input from tributary basins, and the interval soil erosion amount, and then subtracting the sediment output at the lower reaches hydrological station.

**(Ⅴ) Regional sediment output (SD**_**4**_**).** The regional sediment output of the YRB was the sediment transported to the ocean through the Lijin Hydrological Station.

**(Ⅵ) River channel sedimentation (SD**_**5**_**).** The river channel sedimentation was the difference between soil erosion and the sum of sediment deposition from the other four sedimentary processes.

### Estimating carbon emissions caused by soil erosion

Carbon emissions caused by soil erosion are the amount of CO_2_ released from the decomposition of SOC detached and transported by soil erosion processes (Eq. 7):

7 $$\:{C}_{A}={S}_{A}\times\:P\times\:44/12$$

Where *C*_*A*_ is the carbon emissions caused by soil erosion (t); *S*_*A*_ is the total SOC erosion (t); *P* is the decomposed proportion (%) of SOC; 44/12 is the conversion coefficient used to convert carbon into CO_2_. In this study, SOC erosion refers to the loss of SOC resulting from soil erosion processes. Specifically, SOC erosion is estimated as the product of soil loss and SOC content, representing the detachment, mobilization, and redistribution of SOC across the landscape during erosion events.

Equation 6 was inherently scalable and applicable across different spatial and temporal domains, as long as the inputs (e.g., *S*_*A*_ and *P)* can be reasonably estimated. In this study, the method was applied at both the basin scale (entire YRB) and the sub-regional scale (upper and middle reaches), demonstrating its adaptability to different spatial contexts. Account for spatial heterogeneity by using gridded input datasets (e.g., SOC content, soil erosion rates, and sediment deposition) and differentiating sediment redistribution among different depositional environments, including slopes, reservoirs, floodplains, river channels, and regional export. Temporal variability was addressed through the use of multi-year average or time-series input data. Moreover, the framework remains flexible and can be readily adapted to finer temporal resolutions (e.g., seasonal or monthly) if dynamic input data become available.

*P* is calculated based on the study by [39] (Eqs. 8 and 9):

8 $$\:P={S}_{dec}/{S}_{A}\times\:100\mathrm{\%}=\left({S}_{A}-{S}_{dep}\right)/{S}_{A}\times\:100\mathrm{\%}$$

9 $$\begin{aligned}&\:{S}_{dep}\\&\quad=\left({SD}_{1}\times\:{SC}_{1}+{SD}_{2}\times\:{SC}_{2}+{SD}_{3}\times\:{SC}_{3}+{SD}_{4}\times\:{SC}_{4}+{SD}_{5}\times\:{SC}_{5}\right)\\&\qquad\times\:{10}^{3}\end{aligned}$$

where S_*dec*_ is the decomposed SOC (t); *S*_*dep*_ is the deposited SOC (t); *SD*_*1*_, *SD*_*2*_, *SD*_*3*_, *SD*_*4*_, and *SD*_*5*_ are the sediment deposition amounts (t) in slope, reservoirs, plains, regional outputs, and river channels, respectively; *SC*_*1*_, *SC*_*2*_, *SC*_*3*_, *SC*_*4*_, and *SC*_*5*_ are the SOC content (g/kg) in slope, reservoirs, plains, regional outputs, and river channels, respectively.

Due to data limitations, sediment transport and deposition data were only available for 1988–2012. Soil erosion estimates for 1990–2020 were obtained using a five-year moving average approach based on erosion-related factors (e.g., rainfall erosivity), which were continuously available. Due to the lack of sediment observation data from 2012 to 2020, sediment redistribution characteristics and SOC decomposition patterns from 1988 to 2012 were assumed to represent the long-term average for the YRB. Therefore, the decomposition proportion (P) derived from the data from 1988 to 2012 was applied consistently to estimate the carbon emissions resulting from erosion for the entire period from 1990 to 2020.

The total SOC erosion was estimated using spatial distribution data of SOC content. Considering that SOC content varies with soil depth, this study assessed the thickness of the soil erosion layer in the YRB by using soil erosion data and layered spatial distribution data of soil bulk density. Then, the stratified spatial distribution data of soil organic carbon content were used to estimate the loss of total organic carbon. The thickness of the soil erosion layer was calculated as follows (Eqs. 10 and 11):

10 $$\:{A}_{g}=\sum\:_{i=1}^{k}\left({SBD}_{gi}\times\:{T}_{gi}/100\right)\times\:cell\times\:{10}^{-6}$$

11 $$\:{T}_{g}=\sum\:_{i=1}^{k}{T}_{gi}$$

Where *A*_*g*_ is the total soil erosion amounts of grid *g* (t); *i* = 1…*k*, means that the thickness of soil erosion layer was divided into *k* layers; *SBD*_*gi*_ is the soil bulk density of grid *g* (g/m^3^); *T*_*g*_ is the total thickness of soil erosion layer of grid *g*(cm); *T*_*gi*_ is the thickness of soil erosion layer of *i* layer of grid *g* (cm); *cell* is the grid area which is 500 m$\:\times\:$500 m in this study.

The total eroded SOC was calculated as follows (Eq. 12):

12 $$\begin{aligned}&\:{S}_{Ag}=\sum\:_{i=1}^{k}\left({SBD}_{gi}\times\:{T}_{gi}/100\times\:{SC}_{gi}\times\:{10}^{3}\right)\\&\qquad\times\:cell\times\:{10}^{-6}\end{aligned}$$

Where *S*_*Ag*_ is the total eroded SOC of grid *g* (t); *SC*_*gi*_ is the SOC content of the *i* layer of grid *g* (g/kg).

To estimate the decomposed SOC, sediment deposition was simulated using the TLSD model. Then, based on the simulated spatial sediment deposition data, deposition thickness was calculated using Eq. 9, substituting erosion amounts with depositional values. Results showed that in most areas, the calculated sediment deposition thickness was within the range of 0 to 5 cm, with only a few isolated grid cells showing deposition depths between 5 and 15 cm. Therefore, the 0–5 cm surface layer SOC content was used as a representative value for all depositional environments in this study, including slopes and plains. The average SOC content in the Ningxia Plain, Hetao Plain, Fenwei Plain, and Lower Reaches Plain was 0.80%, 0.77%, 0.91%, and 0.89%, respectively. The SOC content of sediment in reservoirs, rivers, and regional outputs was obtained from literature measurements across multiple river sections in the YRB (Table S4).

### Model accuracy verification

This study used the Nash coefficient (*NES*) [44] to verify the accuracy of the CSLE-TLSD model (Eq. 13).

13 $$\:NSE=1-\sum\:_{i=1}^{n}{\left({O}_{i}-{P}_{i}\right)}^{2}/\sum\:_{i=1}^{n}{\left({O}_{i}-\stackrel{-}{O}\right)}^{2}$$

where *n* is observation frequency; *O*_*i*_ and *P*_*i*_ are observed and estimated values, respectively; *O* is average observed value. *NSE*∈[−∞, 1]. The closer the NES value is to 1, the higher the model’s accuracy.

## Results

### Model calibration

This study utilized sediment deposition data from 27 hydrological stations in sub-basins along the YRB from 1988 to 2012 to calibrate the β parameter of the CSLE-TLSD model and validate its predictive accuracy. The β parameter was systematically tested across the range of 1 to 30 with 1-unit increments. Initial calibration revealed that the CSLE-TLSD model achieved its maximum NES of 0.2730 at β = 25, marginally surpassing the baseline CSLE model performance (NES = 0.2682). However, the linear regression analysis between simulated and observed values at this parameter setting exhibited limited correlation strength (R²=0.3737), indicating inconsistencies in model fit across some stations.

Further evaluation identified significant differences at seven hydrological stations (Huangfu, Gaoshiya, Wenjiachuan, Gaojiachuan, Heishiguan, Wuzhi, and Daicunba), where sediment transport rates (i.e., the ratio of sediment generation to model-calculated erosion) deviated significantly from expected ranges. To improve model representativeness for the majority of the basin, these stations were excluded from recalibration. After removal, the optimized β value was moved to 27, which significantly improved the NES to 0.5690 (compared to 0.5628 for the CSLE model) and had a stronger linear correlation (R²=0.6067). Given the improved overall model performance and more reliable simulation accuracy for most hydrological units, the standardized coefficient β = 27 was used in subsequent model applications.

#### Sediment distribution of eroded soils in the YRB region

**Fig. 4:**
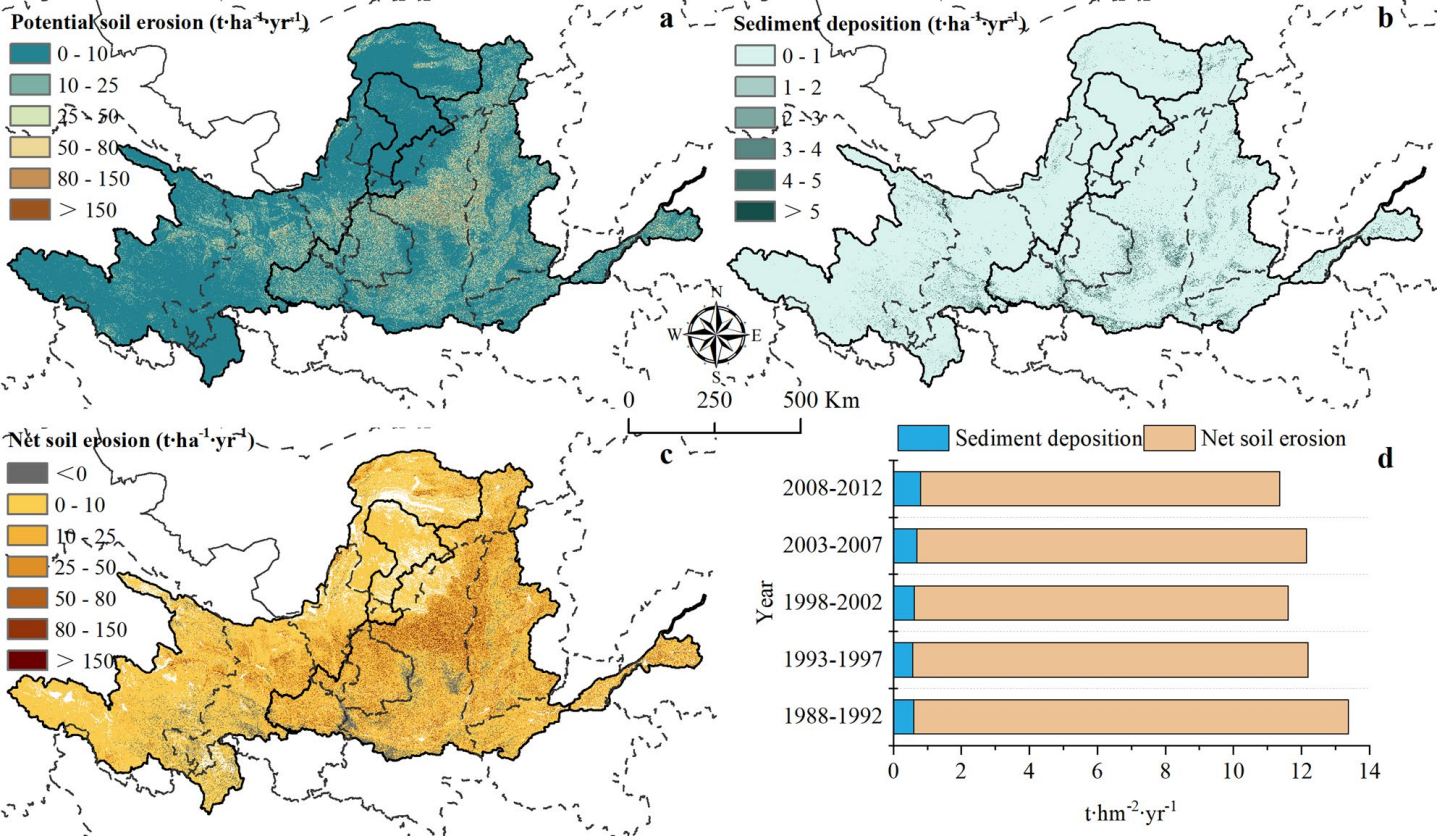
Estimated results in the YRB from 1988 to 2012 based on CSLE-TLSD model. (**a**) Annual average potential soil erosion; (**b**) Annual average sediment deposition; (**c**) Annual average net soil erosion; (**d**) Changing trends in potential soil erosion, sediment deposition, and net soil erosion

From 1988 to 2012, the total potential soil loss was 241.26 × 10^8^ t with the average annual soil erosion modulus of 12.14 t∙hm^−2^∙yr^−1^ in the YRB region (Fig. 4a). Over the study period, the average sediment deposition modulus in the YRB was 0.66 t∙hm^−2^∙yr^−1^ (Fig. 4b). The total slope sedimentation reached 13.02 × 10^8^ t, accounting for 5.40% of the potential soil loss. Among the regions, the total slope sedimentation in the upper and middle reaches was 2.48 × 10^8^ t and 9.85 × 10^8^ t, respectively.

The net soil erosion modulus was 11.48 t∙hm^−2^∙yr^−1^, resulting in a total net soil erosion amount of 228.24 × 10⁸ t (Fig. 4c). Overall, soil erosion in the YRB showed significant improvement during the study period (Fig. 4d). Potential soil erosion exhibited a decreasing trend with a reduction of 15.09%, while sediment deposition increased by 32.47%. As a result, net soil erosion declined markedly by 17.33%.

**Fig. 5:**
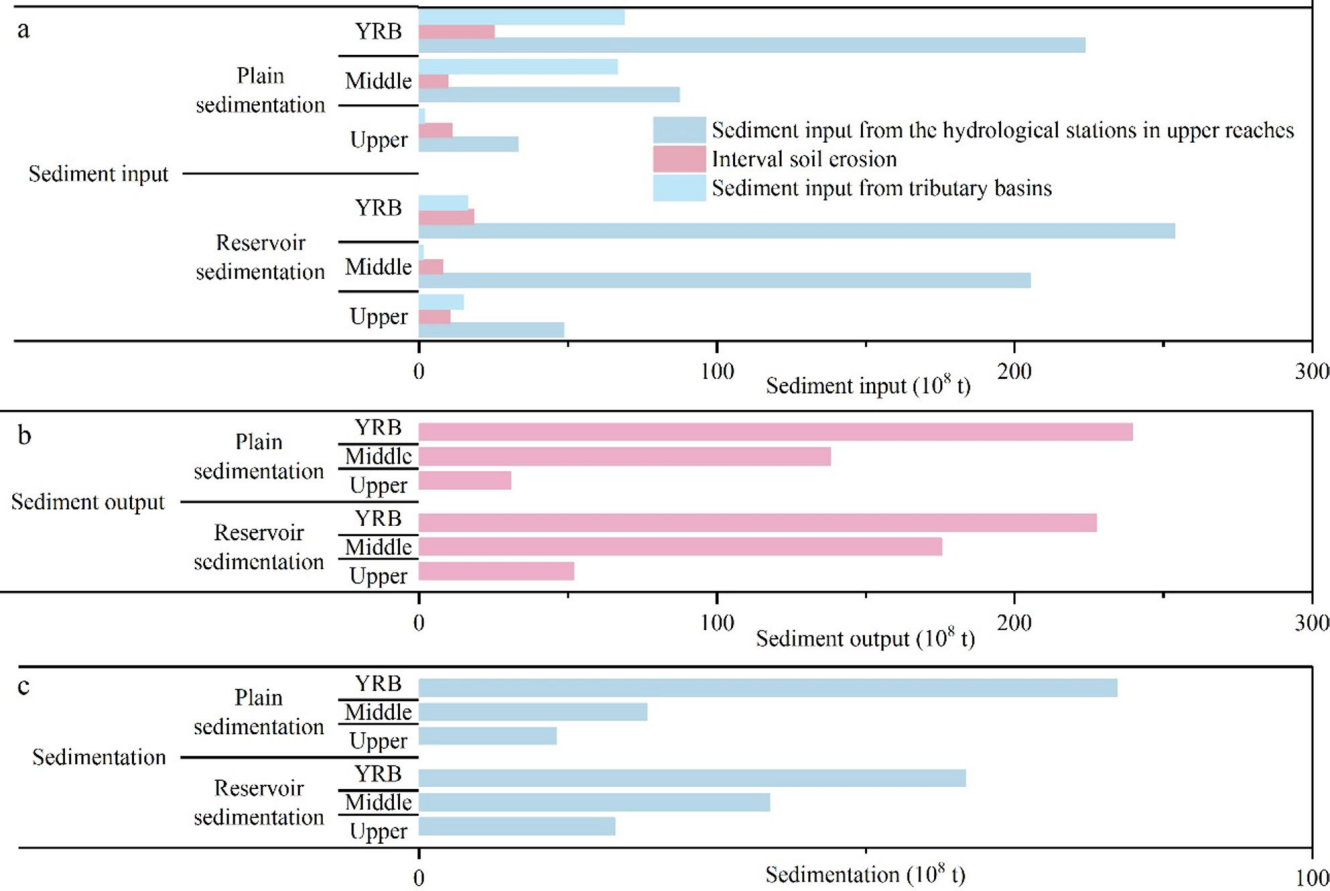
Estimation of reservoir sedimentation and plain sedimentation in the YRB. (**a**) Sediment input from different sources; (**b**) Sediment output of reservoirs and plains; (**c**) Sedimentation in reservoirs and plains

The total sedimentation observed in reservoirs of the YRB reached 61.16 ± 5.80 × 10^8^ t (Fig. 5), accounting for 25.35 ± 2.40% of the potential soil erosion from 1988 to 2012. Of this total, the upper reaches contributed approximately 21.90 ± 0.86 × 10^8^ t while the middle reaches accounted for a substantial 39.26 ± 5.76 × 10^8^ t. Considering that the sediment transported from the upper reaches was also deposited in the reservoirs in the middle reaches, we excluded this part to ensure that the reservoir sedimentation in the middle reaches was entirely supplied by soil erosion within the middle reaches. After adjustment, the total reservoir sedimentation in the middle reach was 27.94 ± 5.76 × 10^8^ t.

During the research period, the sedimentation in the Ningxia Plain, Hetao Plain, Fenwei Plain, and the lower reaches plains were 2.62 ± 0.51 × 10^8^ t、12.77 ± 0.34 × 10^8^ t、25.47 ± 4.62 × 10^8^ t, and 37.27 ± 4.62 × 10^8^ t, respectively, with total sedimentation of 78.13 ± 6.43 × 10^8^ t, accounting for 32.38 ± 2.66% of the total soil erosion in the YRB (Table S3). The plain sedimentation in the upper reaches and middle reaches was 15.39 ± 0.62 × 10^8^ t and 25.47 ± 4.62 × 10^8^ t, respectively (Fig. 5).

The amount of sediment transported from the YRB to the marine system was 70.52 ± 2.27 × 10^8^ t from 1988 to 2012, accounting for 29.23 ± 0.94% of the total soil erosion in the YRB. The regional sediment output for the upper and middle reaches was 11.32 ± 0.23 × 10^8^ t and 102.88 ± 3.82 × 10^8^ t, respectively.

Based on the estimates from the CSLE-TLSD model and regional sediment deposition data, the river channel sedimentation in the YRB amounted to 18.44 ± 8.81 × 10^8^ t from 1988 to 2012, which accounted for 7.64 ± 3.65% of the total soil erosion. The river channel sedimentation for the upper and middle reaches was 8.09 ± 1.08 × 10^8^ and 3.37 ± 8.02 × 10^8^ t, respectively.

### Carbon emissions resulting from soil erosion

#### Estimation of SOC decomposition ratio

The total SOC erosion in the YRB from 1988 to 2012 was estimated at 2.46 × 10^8^ t, calculated by integrating spatially distributed SOC content data with soil erosion derived from the CSLE-TLSD model. Regional analysis revealed distinct contributions: the upper and middle reaches accounted for 0.91 × 10^8^ t (36.99%) and 1.45 × 10^8^ t (58.94%) of the total SOC erosion, respectively.

**Fig. 6:**
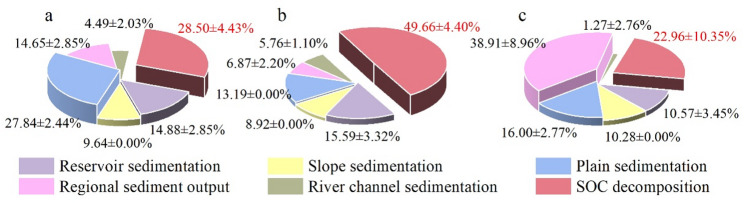
Amount and proportion of organic carbon erosion and redistribution. (**a**) YRB; (**b**) Upper reaches; (**c**) Middle reaches

Obvious spatial patterns of SOC redistribution were detected in different regions of the YRB during the study period. The majority of SOC associated with erosion-derived sediments was deposited in plains, reservoirs, and marine systems, with estimated amounts of 0.68 ± 0.06 × 10^8^ t, 0.37 ± 0.07 × 10^8^ t, and 0.36 ± 0.04 × 10^8^ t, accounting for 27.84 ± 2.44%, 14.88 ± 2.85%, and 14.65 ± 1.63% of the total SOC erosion in the basin, respectively. The total SOC sequestered in sediment deposits was approximately 1.76 ± 0.11 × 10^8^ t. Among them, 0.70 ± 0.11 × 10^8^ t of the mobilized SOC was decomposed and released into the atmosphere as CO_2_ during sediment transport, accounting for 28.50 ± 4.43% of the total eroded SOC (Fig. 6a).

In the upper reaches of the basin, SOC was primarily deposited in reservoir and plain sediments, amounting to 0.14 ± 0.03 × 10^8^ t and 0.12 ± 0.00 × 10^8^ t, respectively, corresponding to 15.59 ± 3.32% and 13.19 ± 0.00% of the total SOC erosion in this region. The total SOC retained in sediments in the upper reaches reached 0.46 ± 0.04 × 10^8^ t, while 49.66 ± 4.40% of the eroded SOC was mineralized and emitted as CO_2_ during transport (Fig. 6b).

In contrast, in the middle reaches, SOC was mainly deposited in regional output and plain sediments, with values of 0.57 ± 0.13 × 10^8^ t and 0.23 ± 0.04 × 10^8^ t, accounting for 38.91 ± 8.96% and 16.00 ± 2.77% of the total SOC erosion in this region, respectively. The total SOC deposition reached 1.12 ± 0.15 × 10^8^ t, with 0.33 ± 0.15 × 10^8^ t being decomposed and released, indicating a decomposition ratio of 22.96 ± 10.35% (Fig. 6c).

#### Estimation of carbon emissions resulting from soil erosion

**Fig. 7:**
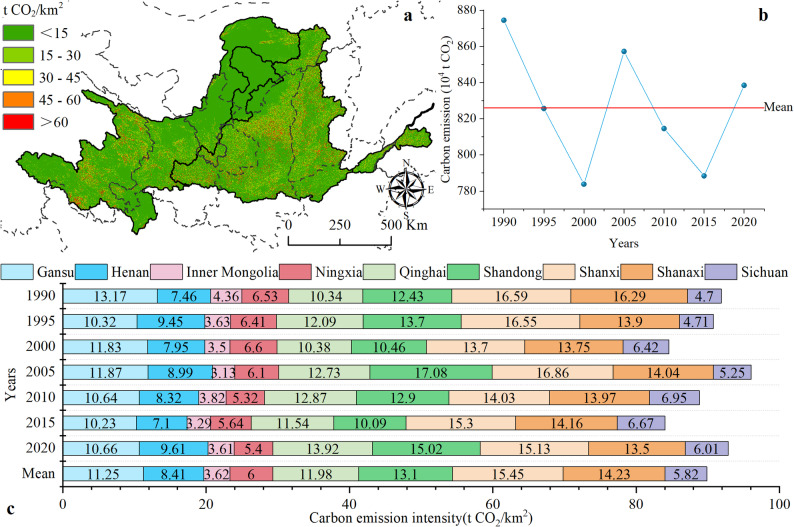
Characteristics of carbon emissions resulting from soil erosion in the YRB from 1990 to 2020. (**a**) Spatial distribution of average annual carbon emissions (t CO_2_/km^2^); (**b**) Change trends of carbon emissions resulting from soil erosion; (**c**) Carbon emission intensity by province (t CO_2_/km^2^)

Considering the unique location of the interflow area of the YRB, the SOC decomposition ratio of 28.50 ± 4.43% was employed to estimate carbon emissions caused by soil erosion. From 1990 to 2020, the average annual carbon emissions in the YRB were approximately 8.26 ± 0.37 × 10^6^ t CO_2_·yr⁻¹, resulting in a cumulative emission of 2.48 ± 0.11 × 10^8^ t CO₂ over 30 years. Significant spatial variation in carbon emissions resulting from soil erosion was detected, with higher emissions in the western of the upper reaches and central regions of the YRB (Fig. 7a). Over the study period, carbon emissions resulting from soil erosion exhibited fluctuations but showed an overall declining trend. Carbon emissions decreased by 4.12% in 2020 compared to 1990 (Fig. 7b).

Significant differences in carbon emissions caused by soil erosion were detected across different provinces (Fig. 7c). The five provinces with the highest carbon emission rate from 1990 to 2020 were Shanxi (15.45 t CO_2_/km^2^), Shaanxi (14.23 t CO_2_/km^2^), Shandong (13.10 t CO_2_/km^2^), Qinghai (11.98 t CO_2_/km^2^), and Gansu (11.25 t CO_2_/km^2^). The carbon emission intensity varies among different provinces. During the study period, the carbon emission intensity of Gansu, Inner Mongolia, Ningxia, Shanxi, and Shaanxi showed a decreasing trend over the three decades, while the carbon emission intensity of Henan, Qinghai, Shandong, and Sichuan continued to rise.

## Discussion

Soil erosion not only causes soil loss but also redistributes SOC and releases CO_2_, significantly contributing to regional carbon emissions. To address this issue, our study combined the CSLE with the TLSD model. This approach simulated the movement of sediment through five key stages: slope, reservoir, river channel, plain sedimentation, and regional sediment output. Crucially, this multi-process framework advanced erosion modeling by accurately tracking sediment fate, particularly in heavily engineered systems like the Xiaolangdi Dam. Our methodology provided a replicable way to assess sediment dynamics in complex river basins with strong erosion and deposition patterns. This research was significant for advancing our understanding of the carbon cycle, informing ecological restoration strategies, and supporting sustainable development goals.

Importantly, in this study, the CSLE-TLSD model was used to comprehensively assess the redistribution of SOC caused by erosion in the YRB, with explicit consideration of various sediment deposition processes. Compared with small-scale studies, the estimated CO_2_ emission rate in this research was relatively low (22.22 gCO_2_·m^− 2^·yr^− 1^), significantly lower than values derived from slope-scale experiments (ranging from 968 to 1338.33 gCO_2_·m^− 2^·yr^− 1^) [45]. This difference can be attributed to the basin-scale analysis approach, which more accurately captures the significant regulatory effects of human interventions such as ecological restoration projects and reservoir operations on sediment and SOC dynamics. Moreover, compared with sediment deposition estimates derived from sediment delivery ratio (SDR) or statistical methods [39, 46], the TLSD model provided more spatially explicit and accurate predictions of sediment redistribution. These strengths make the CSLE-TLSD framework a valuable tool for investigating erosion-induced carbon fluxes across multiple spatial scales and offer robust theoretical support for regional carbon accounting and management.

### Limitations and model uncertainties

The absolute magnitude of carbon emissions resulting from soil erosion was not negligible. Accurate estimation and management of these emissions were crucial for formulating scientific carbon reduction strategies, protecting the ecological environment, and achieving sustainable development. However, this study had some limitations. Firstly, soil erosion was a complex process. While we aimed to account for multiple erosion and deposition stages, the intricate nature of sediment transport may still introduce some uncertainty into the results. For example, although the CSLE-TLSD model accounted for vegetation cover and protective measures through the B (biological practice factor) and E (engineering practice factor, represented by horizontal terraces in this study), it did not explicitly represent the spatial heterogeneity or structural diversity of various vegetative barriers and engineering measures—such as fish-scale pits, check dams, and silt retention basins—that influence sediment retention and redistribution. As a result, the model may underestimate the actual sediment and SOC retention efficiency, particularly in areas where such measures were densely distributed or structurally effective. Future improvements should consider integrating detailed spatial information on the distribution, type, and retention capacity of these vegetative and engineering structures to more accurately capture their mitigating effects on soil erosion and carbon loss. Despite this, the model performance was acceptable, with the NSE of 0.5690 and an R² of 0.6067. Additionally, while soil erosion and deposition alter regional SOC levels, limitations in data availability prevented us from fully accounting for changes in SOC content between 1990 and 2020. This limitation likely affected the accuracy of our estimates of CO_2_ emissions caused by soil erosion. Future studies should incorporate SOC content changed to build upon this work and provide more robust estimates.

Given that the SDR is a critical indicator for evaluating model reliability, hydrological stations with long-term average SDR values exceeding 2 or below 0.1 during the period 1990–2010 were excluded. This resulted in the removal of seven stations to ensure robust model calibration and application across a broader watershed area. Among them, four stations—Huangfu, Gaoshiya, Wenjiachuan, and Gaojiachuan (IDs 25, 27, 28, and 29 in Fig. 2)—are concentrated along the left bank of the Yellow River at the transition between the upper and middle reaches. This region is characterized by highly fragmented terrain and historically intense soil erosion. Between 1990 and 2010, erosion was substantially mitigated through extensive land management efforts, resulting in a 10- to 87-fold reduction in sediment load at these stations. However, the CSLE-TLSD model struggled to reproduce such drastic declines, resulting in high sediment delivery ratios (SDR > 2) and poor model fits. The remaining three stations—Heishiguan, Wuzhi, and Daicunba (IDs 49, 50, and 55 in Fig. 2)—are located in the lower YRB, where flat terrain promotes sediment deposition and results in unusually low sediment output, which also hindered simulation accuracy, with SDR values below 0.1.

### Comparison and mechanistic insights into SOC decomposition and sequestration

Previous studies have suggested that only about 30% of soil eroded from upland areas ultimately reaches the ocean, with the remainder deposited within inland watersheds [47]. Our findings align closely with this understanding, indicating that only 29.23% of total eroded sediment was exported to the ocean between 1990 and 2020, lower than the 36.74% reported by Ran et al. (2014) [39]. This decline reflects increased sediment retention within the basin, driven primarily by the sediment-trapping effects of large reservoirs (e.g., the Xiaolangdi Dam completed in 2000) and extensive soil and water conservation practices implemented since the late 1980s.

The fate of eroded SOC is more uncertain, with the potential for mineralization to CO_2_ during transport [48]. Consistent with earlier studies estimating that 20–40% of eroded SOC undergoes mineralization [7, 49, 50], our simulated decomposition rate of 28.50% is similar to the 27.01% found by Ran et al. (2014) [39]. Moreover, Ran et al. reported an average CO_2_ emission rate of 22.22 g CO_2_·m^−2^·yr^−1^ for 1950–2010, while our simulation yielded 10.39 g CO_2_·m^−2^·yr^−1^ for 1990–2020. Estimates from Yue et al. (2016) for China’s inland sediment transport (19.24 and 15.57 g CO_2_·m^−2^·yr^−1^ for 1995–1996 and 2010–2012, respectively) [46] also fall within a comparable range, reinforcing the reliability of our results despite differences in temporal scope and datasets.

However, the two studies diverge significantly in the burial destinations of non-decomposed SOC. Ran et al. (2014) estimated that 49.53% of eroded SOC was buried within the river system and 23.46% was exported to the ocean. In contrast, our results suggest a pronounced increase in terrestrial SOC sequestration (56.85%), with 27.84% stored in plains and 14.88% retained in reservoirs, while only 14.65% was delivered to the marine system. This shift signifies a fundamental transition in the Yellow River Basin’s carbon sequestration regime—from marine export–dominated in the mid-20th century to land retention–dominated in recent decades—driven by reservoir construction, enhanced plain deposition, and improved soil conservation.

Methodologically, the two approaches also differ. Ran et al. (2014) derived carbon fluxes from a macroscale sediment budget coupled with empirical enrichment ratios, providing a basin-wide but static view of SOC redistribution. By contrast, our process-based, spatially explicit CSLE–TLSD framework dynamically simulates multi-stage sediment transport, deposition, and SOC mineralization, offering a more mechanistic representation of erosion–carbon coupling under contemporary human-modified conditions.

The significant disparity in organic carbon decomposition ratios between the upper (49.66%) and middle (22.96%) reaches partially reflects the regulatory effects of erosional drivers, particularly topography and climate, on carbon cycling dynamics. The type and rate of soil erosion have a significant effect on the amount and nature of SOC transported by soil erosion, and these factors are further shaped by environmental conditions such as topography and climate [48]. Gao, et al. (2018) discovered that cumulative CO_2_ emissions from black soil and loess soil decreased by 38.2% and 10.0% [51], respectively, when rainfall intensity increased from 30 mm∙h⁻¹ to 90 mm∙h⁻¹. When the slope gradient increased from 5° to 25°, cumulative CO_2_ emissions decreased by 23.8% for black soil and 12.6% for loess soil. Du, et al. (2022) regularly monitored the changes in soil CO_2_ emissions in the erosion plots of the Loess Plateau from 2015 to 2019 and found that CO_2_ emissions on the 10° and 20° eroding slopes decreased by 2.8–13.5% and 11.3–15.6% [45], respectively, relative to 5° erosion slopes. These results provide a reasonable explanation for why the upper reaches with lower rainfall and lower slope had a higher decomposition rate relative to the middle reaches with steeper slope and higher rainfall.

### Spatial variations in carbon emissions resulting from soil erosion within the YRB

**Fig. 8:**
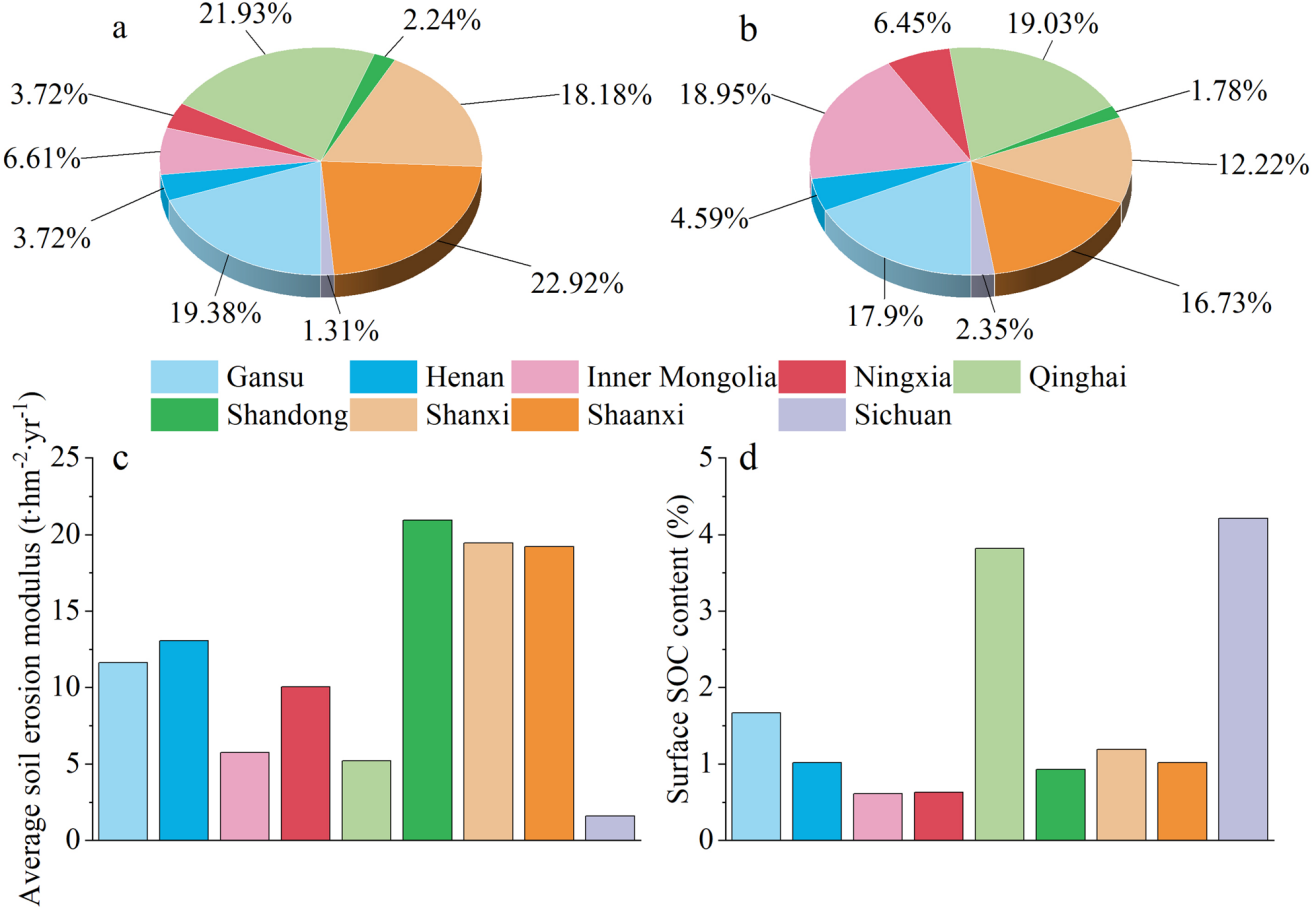
Characteristics of different provinces. (**a**) Proportions of carbon emissions resulting from soil erosion; (**b**) Proportions of area; (**c**) Average soil erosion modulus; (**d**) Average surface SOC content

The higher rates of soil erosion result in lower concentration of C in the transported material because large erosion events have more energy to transport more mineral material, including sediments flushed from deeper soil layers [52]. Therefore, the carbon emissions resulting from soil erosion in Shaanxi (22.92%) and Shanxi (18.18%) provinces had a higher proportion due to the more severe soil erosion (Fig. 8a). However, the carbon emissions resulting from soil erosion in Qinghai and Gansu provinces with relatively mild soil erosion also contribute significantly. To find the reason for the high carbon emissions caused by soil erosion in Qinghai and Gansu, this study further analyzed the main factors affecting soil erosion carbon emissions: soil erosion modulus, area proportion, and surface SOC content. Although the soil erosion modulus in Qinghai (5.21 t∙hm^−2^∙yr^−1^) and Gansu (11.63 t∙hm^−2^∙yr^−1^) were lighter than in Shaanxi and Shanxi (Fig. 8c), their large areas in the YRB (19.03% and 17.90%) (Fig. 8b) and high surface (0–5 cm) SOC content (3.82% and 1.67%) (Fig. 8d) resulted in significant carbon emissions resulting from soil erosion. This also further supports the observation that the SOC decomposition rate was higher in the upper reaches than in the middle reaches.

## Conclusions

In this study, we simulated the sediment redistribution and carbon flux dynamics driven by soil erosion at the basin scale, integrating erosion processes, depositional pathways, and multi-layered SOC data. The calibrated CSLE-TLSD (NSE = 0.569) model was applied to the YRB, and results showed that a total of 2.48 ± 0.11 × 10^8^ t CO_2_ due to the mineralization of SOC from 1990 to 2020, accounting for 28.50 ± 4.43% of the total eroded carbon. Notably, 71.50 ± 6.25% of the mobilized SOC was redistributed across to sedimentary zones, with plains (27.84 ± 2.44%) and reservoirs (14.88 ± 2.85%) acting as critical carbon sinks. The steep upper reaches exhibited a 49.66 ± 4.40% SOC decomposition ratio due to prolonged transport and labile carbon exposure, while middle reaches retained 77.04 ± 13.39% of eroded SOC through rapid deposition in plains and reservoirs.

This study highlights the overlooked role of sediment redistribution caused by erosion as a factor influencing the dynamics of carbon flux within large river basins. By integrating erosion modeling with spatial SOC datasets, we provide precise estimates of erosion-related carbon emissions and identify key sedimentary areas acting as carbon sinks. Although the CSLE-TLSD model enables spatially explicit simulation of erosion-driven carbon loss, limitations remain in fully capturing the mechanistic feedbacks between erosional processes and the carbon cycle. However, our results highlight the need for carbon flux simulations with explicit spatial localization to guide precision land management. Prioritizing erosion mitigation in SOC-vulnerable zones (e.g., upper reaches) and optimizing reservoir sediment retention could enhance carbon sequestration, aligning with Sustainable Development Goal (SDG) targets for climate-resilient basins. Future research should enhance model calibration using site-level sediment and carbon monitoring data, particularly in transitional zones such as middle reaches and reservoirs, and evaluate the impact of climate-driven hydrological extremes on the fate of SOC in erosion-prone landscapes.

## Supplementary Information

Supplementary Material 1

## Data Availability

No datasets were generated or analysed during the current study.
