# Supplementary material for: Quantifying erosion-induced carbon emissions from SOC decomposition across sediment pathways in the yellow river basin
Source: Carbon Balance Manag. 2026 Jan 7;21:45. doi: 10.1186/s13021-025-00380-7 (PMC12930801; doi:10.1186/s13021-025-00380-7)
Supplement: Supplementary file 1 — Supplementary Material 1 [file 13021_2025_380_MOESM1_ESM.docx]

**Quantifying Erosion-Induced Carbon Emissions from SOC Decomposition Across Sediment Pathways in the Yellow River Basin**

Jinwei Guo^a,b^, Yuchun Yang^b^, Mukesh Kumar Soothar^b^, Yanbing Qi^b,^ *

^a^Shanxi Provincial Department-Municipal Key Laboratory Cultivation Base for Quality Enhancement and Utilization of Shangdang Chinese Medicinal Materials, School of Pharmacy, Changzhi Medical College, Changzhi, Shanxi, 046000, China

^b^College of Natural Resources and Environment, Northwest A&F University, Yangling, Shaanxi, 712100, China

**E-mail addresses:**

**Corresponding author:** ybqi@nwsuaf.edu.cn (Y. Qi)

**Authors:** [guojinwei@nwafu.edu.cn](mailto:guojinwei@nwafu.edu.cn) (J. Guo); [18763890168@163.com](mailto:18763890168@163.com) (Y. Yang); [mukeshksootar@gmail.com](mailto:mukeshksootar@gmail.com) (M. Kumar)

**Table. S1** Hydrological stations and their control area

| Control area | Hydrological stations | Control area | Hydrological stations |
| --- | --- | --- | --- |
| 1 | Heyuan—Huangheyan | 30 | Shenjiawan |
| 2 | Huangheyan—Jimai | 31 | Linjiaping |
| 3 | Jimai—Maqu | 32 | Fugou—Gaoshiya—Wenjiachuan—Gaojiachuan—Shenjiawan—Linjiaping—Wubo |
| 4 | Maqu—Tangnaihai | 33 | Houdacheng |
| 5 | Tangnaihai—Guide | 34 | Baijiachuan |
| 6 | Guide—Xunhua | 35 | Yanchuan |
| 7 | Hongqi | 36 | Daning |
| 8 | Xunhua—Hongqi—Xiaochuan | 37 | Gangguyi |
| 9 | Xiaochuan—Shangquan | 38 | Dacun |
| 10 | Minhe | 39 | Jixian |
| 11 | Xiangtang | 40 | Wubo—Baijiachuan—Yanchuan—Ganggu |
| 12 | Shangquan—Xiangtang—Minhe—Lanzhou | 41 | Hejin |
| 13 | Jingyuan | 42 | Zhangjiashan |
| 14 | Lanzhou—Jingyuan—Anningdu | 43 | Xianyang |
| 15 | Anningdu—Xiaheyan | 44 | Xianyang—Zhangjiashan—Huaxian |
| 16 | Quanyanshan | 45 | Zhuangtou |
| 17 | Xiaheyan—Quanyanshan—Qingtongxia | 46 | Longmen—Zhuangtou—Huaxian—Tongguan |
| 18 | Guojiaqiao | 47 | Tongguan—Sanmenxia |
| 19 | Qingtongxia—Guojiaqiao—Shizuishan | 48 | Sanmenxia—Xiaolangdi |
| 20 | Shizuishan—Dengkou | 49 | Heishiguan |
| 21 | Dengkou—Bayangaole | 50 | Wuzhi |
| 22 | Bayangaole—Sanhuhekouw | 51 | Xiaolangdi—Heishiguan—Wuzhi—Huayuankou |
| 23 | Sanhuhekouw—Toudaguai | 52 | Huayuankou—Jiahetan |
| 24 | Pianguan | 53 | Jiahetan—Gaocun |
| 25 | Huangfu | 54 | Gaocun—Sunkou |
| 26 | Toudaguai—Huangfu—Pianguan—Fugou | 55 | Daicunba |
| 27 | Gaoshiya | 56 | Sunkou—Daicunba—Aishan |
| 28 | Wenjiachuan | 57 | Aishan—Luokou |
| 29 | Gaojiachuan | 58 | Luokou—Lijin |

**Table. S2** The commissioning date of reservoirs

| Reservoir name | Commissioning date |
| --- | --- |
| Bnaduo Reservoir | October 2010 |
| Longyangxia Reservoir | October 1986 |
| Laxiwa Reservoir | March 2009 |
| Lijiaxia Reservoir | October 1991 |
| Zhiganglaka Reservoir | May 2005 |
| Kangyang Reservoir | October 2005 |
| Gongboxia Reservoir | August 2004 |
| Suzhi Reservoir | September 2005 |
| Huangfeng Reservoir | September 2015 |
| Liujiaxia Reservoir | October 1968 |
| Yanguoxia Reservoir | March 1961 |
| Xiaoxia Reservoir | November 2002 |
| Daxia Reservoir | November 1996 |
| Wujinxia Reservoir | 2008 |
| Qingtongxia Reservoir | December 1967 |
| Sanshenggong Water Conservancy Hub | May 1961 |
| Wanjiazhai Reservoir | October 1998 |
| Longkou Reservoir | September 2009 |
| Sanmenxia Reservoir | April 1957 |
| Xiaolangdi Reservoir | October 1997 |
| Xixiayuan Counter Regulating Reservoir | June 2007 |

**Table S3**. Estimation of reservoir sedimentation and plain sedimentation in the YRB (10^8^ t)

| Type | Zone | Control area of hydrological station / Plain | Time periods | Sediment input from the hydrological stations in upper reaches | Interval soil erosion | Sediment input from tributary basins | Sediment output at the hydrological station in lower reaches | Sedimentation |
| --- | --- | --- | --- | --- | --- | --- | --- | --- |
| Reservoir sedimentation | Upper reaches | 4 | 2011-2012 | 0.11±0.02 | 0.26 | 0.00 | 0.25±0.07 | 0.12±0.07 |
|  |  | 5 | 1988-2012 | 2.72±0.08 | 2.60 | 0.00 | 0.61±0.03 | 4.70±0.09 |
|  |  | 6 | 1992-2012 | 0.42±0.01 | 2.05 | 0.00 | 2.14±0.09 | 0.33±0.09 |
|  |  | 8 | 1988-2012 | 2.94±0.12 | 2.27 | 3.80±0.11 | 3.79±0.11 | 5.22±0.20 |
|  |  | 9 | 1988-2012 | 3.79±0.11 | 0.42 | 0.00 | 4.01±0.12 | 0.20±0.16 |
|  |  | 14 | 1997-2012 | 3.20±0.09 | 1.93 | 3.19±0.15 | 7.93±0.29 | 0.40±0.34 |
|  |  | 17 | 1988-2012 | 16.97±0.36 | 0.76 | 7.87±0.25 | 17.92±0.40 | 7.68±0.59 |
|  |  | 21 | 1988-2012 | 18.50±0.34 | 0.14 | 0.00 | 15.39±0.25 | 3.25±0.42 |
|  |  | Total | - | 48.65±0.54 | 10.43 | 14.86±0.31 | 52.04±0.59 | 21.90±0.86 |
|  | Middle reaches | 26 | 1999-2012 | 6.12±0.18 | 4.97 | 1.36±0.10 | 2.68±0.12 | 9.77±0.24 |
|  |  | 47 | 1988-2012 | 138.27±3.59 | 1.81 | 0.00 | 149.15±3.73 | -9.07±5.18 |
|  |  | 48 | 1998-2012 | 56.65±1.86 | 1.02 | 0.00 | 18.63±1.55 | 39.06±2.42 |
|  |  | 51 | 2007-2012 | 4.16±0.48 | 0.22 | 0.07±0.01 | 4.95±0.42 | -0.50±0.64 |
|  |  | Total | - | 205.21±4.08 | 8.02 | 1.43±0.10 | 175.41±4.06 | 39.26±5.76 (27.94±5.76) |
|  | Total in the YRB | | - | 253.86±4.11 | 18.46 | 16.29±0.33 | 227.45±4.08 | 61.16±5.80 |
| Plain sedimentation | Upper reaches | Ningxia Plain | - | 17.92±0.40 | 2.28 | 1.87±0.09 | 19.44±0.31 | 2.62±0.51 |
|  |  | Hetao Plain | - | 15.39±0.25 | 8.70 | 0.00 | 11.32±0.23 | 12.77±0.34 |
|  | Total | | - | 33.31±0.47 | 10.98 | 1.87±0.09 | 30.76±0.39 | 15.39±0.62 |
|  | Middle reaches | Fenwei Plain | - | 87.47±2.58 | 9.60 | 66.67±1.33 | 138.27±3.59 | 25.47±4.62 |
|  | Lower reaches | Lower Reaches Plain | - | 102.87±3.82 | 4.57 | 0.33±0.02 | 70.52±2.27 | 37.27±4.44 |
|  | Total in the YRB | | - | 223.65±4.63 | 25.15 | 68.87±1.33 | 239.55±4.26 | 78.13±6.43 |

Note: Due to significant sediment accumulation, the water level of Sanmenxia Reservoir and Xiaolangdi Reservoir has risen. These two reservoirs have been clearing the sediment for a long time, which has led to a high sediment output at the hydrological stations in the lower reaches of the reservoirs, so the sedimentation of some reservoirs has shown negative values.

**Table S4.** The SOC content and sources of different redistribution items

| Area | SOC redistribution items | SOC content in this study (%) | SOC content in papers (%) | Sources | Note |
| --- | --- | --- | --- | --- | --- |
| Upper reaches | River channel and reservoir sedimentation | 0.645±0.118 | 0.44-0.85 | [1] | The main river channel downstream of the Lanzhou hydrological station |
|  | Regional sediment output | 0.55±0.13 | 0.4-0.8 | [2] | The middle and lower reaches of the YRB |
| Middle reaches | River channel and reservoir sedimentation, regional sediment output |  | 0.11-0.89 | [3] | Weekly sampling data from the Toudaguai hydrological station to the Lijin hydrological station |
| YRB | River channel sedimentation and regional sediment output | 0.5975±0.0878 | - | - | Average values for the upper and middle reaches of the YRB |
|  | Regional sediment output | 0.51±0.05 | 0.4-0.6 | [4] | Sedimentation at Lijin hydrological station (< 16 µm) |
|  |  |  | 0.37-0.79 | [5] | Monthly sampling data at the Lijin hydrological station |
|  |  |  | 0.42-0.5 | [6] | Sediments near the Yellow River estuary |

**References**

1.Liu, D.; Zhang, L., Temporal and Spatial Distributions of Organic Carbon in the Huanghe (Yellow) River. Periodical of Ocean University of China. 2010; 40: 105-110. <https://https://10.16441/j.cnki.hdxb.2010.12.015>.

2.Wang, H.; Yang, Z.; Saito, Y.; Liu, J. P.; Sun, X.; Wang, Y., Stepwise decreases of the Huanghe (Yellow River) sediment load (1950–2005): Impacts of climate change and human activities. Global Planet Change. 2007; 57: 3: 331-354. <https://https://doi.org/10.1016/j.gloplacha.2007.01.003>.

3.Ran, L.; Lu, X. X.; Xin, Z.; Yang, X., Cumulative sediment trapping by reservoirs in large river basins: A case study of the Yellow River basin. Global Planet Change. 2013; 100: 308-319. <https://https://doi.org/10.1016/j.gloplacha.2012.11.001>.

4.Zhang, L.; Zhang, J.; Gong, M., Size distributions of hydrocarbons in suspended particles from the Yellow River. Appl Geochem. 2009; 24: 7: 1168-1174. <https://https://doi.org/10.1016/j.apgeochem.2008.12.033>.

5.Wang, X.; Ma, H.; Li, R.; Song, Z.; Wu, J., Seasonal fluxes and source variation of organic carbon transported by two major Chinese Rivers: The Yellow River and Changjiang (Yangtze) River. Global Biogeochem Cycles. 2012; 26: 2. <https://https://doi.org/10.1029/2011GB004130>.

6.Cauwet, G.; Mackenzie, F., Carbon inputs and distribution in estuaries of Turbid Rivers - The Yangtze and Yellow Rivers (China). Marine Chemistry - MAR CHEM. 1993; 43: 235-246. <https://10.1016/0304-4203(93)90229-H>.
